# Persistent Breeding-Induced Endometritis in Mares—A Multifaceted Challenge: From Clinical Aspects to Immunopathogenesis and Pathobiology

**DOI:** 10.3390/ijms21041432

**Published:** 2020-02-20

**Authors:** Igor F. Canisso, Lorenzo G.T.M. Segabinazzi, Carleigh E. Fedorka

**Affiliations:** 1 Department of Veterinary Clinical Medicine, College of Veterinary Medicine, University of Illinois Urbana-Champaign, Champaign, IL 61802, USA; lgseg@hotmail.com; 2 Department of Animal Reproduction and Veterinary Radiology, Faculty of Veterinary Medicine and Animal Science, São Paulo State University (UNESP), Botucatu 18618-000, São Paulo, Brazil; 3 The Maxwell H. Gluck Equine Research Center, University of Kentucky, Lexington, KY 40503, USA; carleigh.fedorka@uky.edu

**Keywords:** subfertility, uterine infection, horses, inflammation, endometrium

## Abstract

Post-breeding endometritis (i.e., inflammation/infection of the endometrium), is a physiological reaction taking place in the endometrium of mares within 48 h post-breeding, aimed to clear seminal plasma, excess sperm, microorganisms, and debris from the uterine lumen in preparation for the arrival of an embryo. Mares are classified as susceptible or resistant to persistent breeding-induced endometritis (PBIE) based on their ability to clear this inflammation/infection by 48 h post-breeding. Mares susceptible to PBIE, or those with difficulty clearing infection/inflammation, have a deficient immune response and compromised physical mechanisms of defense against infection. Molecular pathways of the innate immune response known to be involved in PBIE are discussed herein. The role of the adaptive uterine immune response on PBIE remains to be elucidated in horses. Advances in the pathobiology of microbes involved in PBIE are also revised here. Traditional and non-traditional therapeutic modalities for endometritis are contrasted and described in the context of clinical and molecular aspects. In recent years, the lack of efficacy of traditional therapeutic modalities, alongside the ever-increasing incidence of antibiotic-resistant microorganisms, has enforced the development of non-traditional therapies. Novel biological products capable of modulating the endometrial inflammatory response are also discussed here as part of the non-traditional therapies for endometritis.

## 1. Introduction

Endometritis, infection and/or inflammation of the endometrium, is the number one cause of subfertility and the third most common disease affecting horses [1,2]. Clinically, mares are classified as susceptible or resistant to persistent breeding-induced endometritis (PBIE) based on their ability to clear this inflammation/infection by 48 h post-breeding [3,4]. Susceptible mares, or those with difficulty clearing inflammation, may have a poor vulvar conformation in addition to a pendulous uterus [5]. Additionally, susceptible mares are typically older [6]. In a normal breeding program, as many as 25% of broodmares are ≥ 16 years old, which is when the fertility of mares starts to decline [7]. It is estimated that 10–15% of mares are susceptible to developing PBIE [8].

A normal phenomenon, post-breeding inflammation can be caused by infectious agents (i.e., bacteria and fungus) and or by non-infectious agents such as sperm [9]. All mares display this transient uterine inflammatory response within 30 minutes following natural mating or artificial insemination [10,11,12]. This physiological reaction occurs to eliminate seminal plasma, excess sperm, microorganisms, and debris from the uterine lumen in preparation for the arrival of an embryo [9]. However, mares deemed susceptible to PBIE have a delayed onset followed by a prolonged uterine inflammatory response [12,13,14], which culminates with excessive accumulation of polymorphonuclear neutrophil (PMNs) and intrauterine fluid accumulation in the uterus up to 96 h and beyond after breeding, impairing embryonic survival and the establishment of pregnancy [8,15,16]. Additionally, due to impaired innate immune response activation, microbes introduced into the uterus at the time of breeding are not eliminated efficiently, leading to potential infection.

The management of mares susceptible to PBIE is a daunting task. The equine embryo migrates from the uterine tube to the uterine lumen between 144 and 168 h after ovulation [17]. This occurs concomitantly with the increasing production of progesterone and cervical tone, leading to minimal time to resolve inflammation. Persistent neutrophilia, excess intraluminal fluid accumulation, and prolonged pro-inflammatory cytokine production are all embryotoxic and lead to decreased fertility potential of these mares in either natural mating or artificial insemination [18]. Additionally, embryo donor mares are bred and flushed multiple times per season, for cumulative breeding seasons, making them particularly prone to endometritis [19]. The persistent endometrial inflammation creates a hostile uterine environment for the embryo entering the uterus, compromising embryo recovery and pregnancy rates [12,13].

Traditionally, endometritis has been treated with multi-modal therapeutics such as a combination of uterine lavage, ecbolic agents, anti-inflammatories, and antibiotics. Unfortunately, a subset of mares fails to respond to traditional therapeutics [20,21]. The lack of efficacy to traditional therapeutics, alongside the ever-increasing incidence of antibiotic-resistant microorganisms, has led to the development of alternative therapies for mares suffering from PBIE [21].

While equine endometritis has been a widely studied disease in the past 40 years and elegant reviews have been published on the subject [22,23,24,25,26,27], much progress has been made in the last decade towards the understanding of the molecular aspects involved in PBIE and pathways down- or up-regulated in response to experimental induction and treatment [17,28,29,30,31,32,33,34,35]. Therefore, this manuscript aims to revise the clinical, molecular, and microbiological aspects of the pathogenesis, diagnosis, and treatment of endometritis in mares.

## 2. Etiology and Pathogenesis of Endometritis

Endometritis can be divided into both infectious and non-infectious causes, and yet in clinical practice, they often occur in association with another. The clinical signs for these two types of endometritis can be indistinguishable, except that the first type has microorganism(s) involved. In addition, to date, there are limited controlled studies comparing infectious vs. non-infectious endometritis in mares [36,37]. Mares with defective reproductive anatomy (e.g., poor vulvar conformation, torn vestibulovaginal sphincter, ventral sacculation of the uterus, impaired uterine contractility, cervix incompetence, and atrophied endometrium folds) are more prone to aspirate air or accumulate fluid or urine in the vagina and uterus, which make the mare simultaneously prone to infectious and non-infectious endometritis [20,38]. Additionally, mares with competent immune response and functional anatomy of the reproductive tract are able to clear infections spontaneously (i.e., mares resistant to endometritis), whereas mares with a deficient immune response may be unable to combat the development of an infection or may have persistent inflammation [31,32,33,39].

### 2.1. Infectious Endometritis

Infectious endometritis plays a major role in equine subfertility [40,41]. Microorganisms, including pathogenic or opportunistic bacteria and fungi, may gain access to the uterus during breeding. While the resistant mare should rapidly respond to the presence of microorganisms, inadequate immune response, and impaired uterine fluid drainage (e.g., due to pendulous uterus, tight cervix, or impaired myometrial contractility) may lead to infection [42,43]. Endometritis is mostly associated with aerobic bacteria [40]; however, anaerobes may also invade the uterus [44]. Of interest, retrospective reports identified that 25-60% of mares failing to become pregnant have bacterial uterine infection [45,46]. In clinical cases, the most commonly isolated bacteria linked with endometritis are the *Streptococcus* species, followed by Coliforms, *Pseudomonas aeruginosa*, and *Staphylococcus aureus* [20,42,47,48,49] (Table 1). Among all, *Streptococcus equi* subspecies *zooepidemicus* (*Streptococcus zooepidemicus*) and *Escherichia coli* predominate as causes of acute and chronic endometritis, respectively [50,51]. It is worth noting that *Streptococcus zooepidemicus* has also been shown to cause dormant, deep-seeded infections in the endometrium of mares, making them resistant to traditional therapy [52].

Fungi are less commonly associated with endometritis (1–5%), and they may occur alone or in association with bacteria [54]. *Aspergillus* and *Candida* are the most common genera, but other species have also been less commonly identified (e.g., *Mucor* sp) [49,53,54] (Table 1). It should be noted that fungal endometritis typically occurs as an opportunistic infection and has been identified after repeated use of intrauterine antimicrobials [53,55,56].

Mares susceptible to PBIE are prone to developing chronic infections, and some of those infections are due to bacteria and fungi capabilities to produce biofilm [57,58,59]. Biofilm is a complex aggregate of microorganisms and their secretions (i.e., extracellular matrix of polymeric substances) [60], which confers the ability to microorganisms to evade the immune system [61,62,63,64]. Biofilm works as a barrier for the diffusion of antimicrobials, and this limited penetration results in resistance to antimicrobial therapy, particularly when compared with planktonic bacterial infection (bacteria without biofilm) [65,66,67].

The shift of bacteria from the planktonic to the biofilm stage is attained via bacterial cell signaling molecule cyclic di-GMP [68,69], which regulates the production of exopolysaccharides alginate, Pel and Psl [70,71,72]. It is worth noting that both Pel and Psl are involved in the attachment of bacteria to a cellular or a noncellular substrate and in the attachment of a microcolony to a substrate and stabilization of extracellular DNA to support the biofilm [73,74,75,76,77]. Approximately 80% of bacteria isolated from the equine uterus is capable of producing a biofilm [58,59,78,79]. The host immunity and microenvironment are known to play a role in biofilm formation in other body systems such as the oral cavity [80,81]; however, it remains to be determined how these factors contribute to biofilm formation and pathogenesis of endometritis in the equine uterus [58,78,82].

Previously, it was believed that the mammalian female uterine environment was sterile [83,84,85]; however, this claim was challenged after the publication of the Human Microbiome Project (2007), which showed that the uterine cavity harbors a unique microbiome [83,85,86]. In horses, the uterus supports a moderately diverse microbiome, and its composition appears largely shared with microbial populations found on the external cervical *os* [87]. This communality of microbe populations between the cranial vagina and uterus in mares can be explained by the open cervix and close communication between the uterine lumen and cranial vagina during estrus [88]. The uterine microbiome changes according to the stage of the estrous cycle and across studies [89,90]. In one study [87], Proteobacteria-driven microbiota was the primary population, while a more diverse microbiome, including Proteobacteria, Firmicutes, Bacterioidetes, and Actinobacteria, was reported in a different study [91].

The uterine microbiome of women and cows suffering from endometritis differs from that of healthy females [92], suggesting that endometritis is associated with commensal microbiome dysbiosis [88]. Invasion of the uterine cavity in mares and other mammals mostly occurs via ascending migration from the vagina [86,93], in association with one or more reproductive problems as described above. The role of the resident uterine microbiome in preventing infection and its potential interactions with the embryo and potential role on pregnancy loss have not been elucidated in horses. Similarly, the role of therapies for uterine infections in restoring a healthy balance in the uterine microbiome has not been studied in horses.

### 2.2. Non-Infectious Endometritis

In the past, endometritis was believed to be caused solely by a bacterial or fungal infection. Yet, pioneer studies found a similar neutrophil response regardless of if the mare was challenged with sperm, saline, or bacteria [12,37,94]. The immune system of the mucosal reproductive tract consists of two branches: the innate and the adaptive. Adaptive immunity responds selectively and gradually to the detection of antigens, and this is mediated by the T lymphocytes [95]. In contrast, the response to breeding is dictated by the innate immune response [28,30]. This consists of a non-specific, rapid, and transient response [96]. Initiated by Toll-like receptors (TLRs), immunoglobulins, and complement, it leads to the leukocytic digestion and elimination of foreign material regardless of pathogenicity [97,98,99]. The predominantly innate immune response allows for repeat spermatozoic and embryonic “challenge” to the immune system without the development of anti-sperm or anti-embryo antibodies. More specifically, regulatory T cells (Treg cells) recognize male antigens and develop a tolerogenic immune environment by suppression of inflammation and immune rejection responses [100,101]. Treg cells are a population of T lymphocyte with immune suppressive properties that comprise both CD4+ and CD8+ subtypes [102]. Treg cells generally act to suppress cytokine synthesis and effector function in macrophages, T-, B-, natural killer (NK), and dendritic cells [103,104].

In addition, Treg cells play a paramount role in mediating the immune tolerance required for embryo implantation by increasing circulating CD4+ CD25+ cells highly enriched with the signature Treg transcription factor forkhead box protein P3 (FoxP3) [100,105,106]. It is worth noting that FoxP3 was identified as a potent marker for Tregs in mice; it is also essential for the immunosuppressive action of Treg cells [107,108]. During early normal pregnancy, there is a decrease in CD4+/CD8+ ratio and an increment of CD4+CD25+FoxP3 in the mouse model [107,108,109]. In addition, Treg-related transcripts are increased during pregnancy in comparison with non-pregnant diestrus mares, and FoxP3-positive cells in the fetoplacental unit are associated with pregnancy and gestational age in mares [110]. However, it remains to be determined if susceptibility to PBIE is also associated with a disruption of these physiological mechanisms in mares and consequent compromise the establishment of a pregnancy. Soluble factors, including transforming growth factor-β, prostaglandin E, and TLR4 ligands, which are present in male seminal fluid, also contribute to an immune-deviating activity that drives to the development of this tolerogenic immune environment *in vivo* mice and *in vitro* human models [100,111,112]. Anecdotally, it has been long speculated in clinical practice that mares bred to the same stallion repeatedly multiple times per season, for cumulative breeding seasons, particularly in embryo transfer programs, are prone to develop more profound PBIE or not to become pregnant; however, satisfactory pregnant rates can be achieved when these mares are bred to males not previously used for breeding. The disruption of some of these physiological pathways may alter the uterine immune response after repeated exposure to the same male; however, these hypotheses have not been critically addressed in horses.

### 2.3. Innate Immune Response to Endometritis

The local innate immune response is immediately activated after antigen recognition and signaling by mucosal epithelial cells in the endometrium [28,30]. The detection of foreign particles induces the activation of the innate immune system, which is the first line of defense. The major functions of the innate immune system are to (1) recruit immune cells to sites of infection through the activation of various cytokines including chemokines, (2) activate the complement cascade to promote the clearance of dead cells, (3) induce the activation of the adaptive immune system through antigen presentation, and (4) act as a physical barrier to the invading organisms and particles. Many of these functions have been studied for their involvement in the disorder of endometritis, a review of which is provided here.

**Figure 1:**
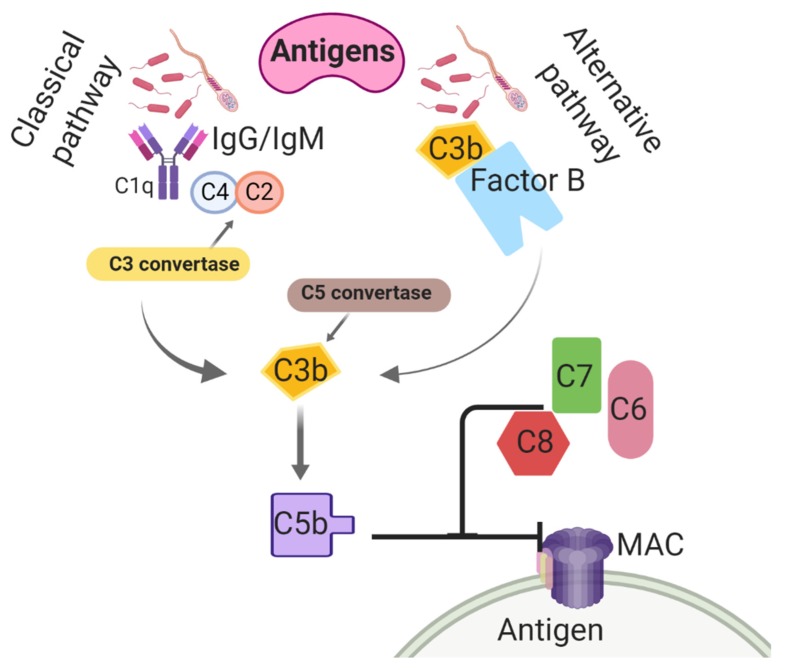
Classical and alternative complement activation pathways taking place in the uterine lumen of mares post-breeding. C1q: Complement component 1; C2: Complement 2; C3: Complement 3; C4: Complement 4; C5: Complement 5; C6: Complement 6; C7: Complement 7; C8: Complement 8; IgG: immunoglobulin G; IgM: immunoglobulin M; MAC: membrane attack complex.

Believed to be the most ancient arm of the immune system, the complement system is utilized to nonspecifically enhance the phagocytic clearance of damaged cells and microbes [113]. Complement is an important contributor to opsonization activity in uterine secretions and inflammatory cell chemotaxis, preceding an inflammatory response [114]. In the classical complement pathway, the C4 subunit binds to IgM/IgG-associated C1q, initiating the enzymatic cleavages of C4 into C4a and C4b and C2 into C2a and C2b [115] (Figure 1). The association of C4b with C2b activates C3 convertase. Thereafter, C3 convertase cleaves C3 into C3a, and C3b [116] and then C3a contributes to leukocyte recruitment and further complement activation [117,118]. In addition, C3b binding to C4b activates convertase [119,120]. Both C4b and C3b are then able to bind to microbe surfaces or additional immunoglobulins [121]. The C5 convertase breaks C5 into C5a and C5b, and this results in conformational changes and activation of the terminal pathway, which promotes the formation of the membrane attack complex (MAC) [122]. The MAC is synthesized by interactions between C5b and other terminal components (e.g., C6, C7, C8, and C9) and lyses the target cell(s) by creating pores in the plasma membrane [123]. The complement cascade can also be triggered by an alternative pathway, which entails C3b protein binding directly to antigens [124]. Interestingly, the activation of this system has been found in response to breeding in the horse. Equine spermatozoa were found to induce the complement cascade, resulting in an increase of C3b and C5a, leukotrienes, and prostaglandins, all of which result in the chemotaxis of PMNs to the uterus [125,126,127].

Additionally, complement cleavage factor C3b has been found in uterine secretions in both resistant and susceptible mare populations [128,129,130]. The various classes of immunoglobulins (e.g., IgA, IgG, and IgM) have also been identified in the uterine secretions of the mare. In addition to their relationship to complement, these molecules are thought to play an important role in antigen presentation to T cells [131,132,133,134,135]. Interestingly, no differences have been found when comparing the resistant mare to the susceptible mare regarding the expression or production of the various complement subunits or immunoglobulins, suggesting that they play a limited role in the pathogenesis of susceptibility to PBIE [2,136].

Microbes and foreign particles (such as sperm and seminal plasma proteins) are also detected via their antigen presentation to pattern recognition receptors (PRRs) located in the endometrial epithelial cells [29,137,138]. These cells, alongside the immune cells (e.g., tissue macrophages, NK cells, and neutrophils) that they recruit, produce various types of cytokines, including chemokines. Chemokines further recruit leukocytes to the site of inflammation, while other cytokines enable the differentiation and activation of other chemotaxed immune cells [139,140]. Collectively, these cells form a physical and immunological barrier at the uterine mucosa [141].

Toll-like receptors, a family of transmembrane proteins expressed in mammal cells [142,143,144,145], play a major role in that antigen recognition [146]. In this role, antigen-presenting cells (ACP), mainly dendritic, macrophages, and NK cells, express molecules to recognize these patterns [97,147]. Pathogen-associated molecular patterns (PAMPs) are recognized by the TLRs of endometrial and sentinel cells to begin the inflammatory reaction [148]. The exposure of Gram-negative bacteria or lipopolysaccharides (LPS) to these cells has been shown to enhance the expression of TLRs types 2 and 4 in mice, rabbits, and cattle [61,62,63,64]. The expression of TLRs by the endometrium after interaction with sperm is still not fully elucidated, particularly because semen is not sterile, and inherent bacterial contamination can be responsible for conflicting results in the literature. Toll-like receptor type 4 primarily recognizes LPS produced by Gram-negative bacteria, while TLR2 reacts to lipopeptides of Gram-positive bacteria, which may invade the uterus during breeding [149,150]. It is known that TLR2 and TLR4 are increased in the uteruses of mares resistant to PBIE after inoculation with *Escherichia coli* [29]. Additionally, work in other species indicates that human and murine sperm express TLR2 and TLR4, although this has not been confirmed in the horse [151]. In bovine, *in vitro* studies suggested that sperm can stimulate transcription of pro-inflammatory cytokines, including chemokines (TNFα, IL1β, CXCL8) and prostaglandin E and activate the local complement system (C3) mediated via TLR2/4 [152,153,154].

Another group of PRRs is NOD-like receptors (NLR), which are responsible for the intracellular detection of pathogens [155]. NOD-like receptors are expressed in many cell types, including immune cells and epithelial cells, although certain NLR family members are expressed primarily in phagocytes, including macrophages and neutrophils. A genetic variation in these genes may predispose humans to several inflammatory diseases [155,156]; however, the role of NLRs on the pathogenesis of endometritis has not been elucidated in horses.

**Figure 2:**
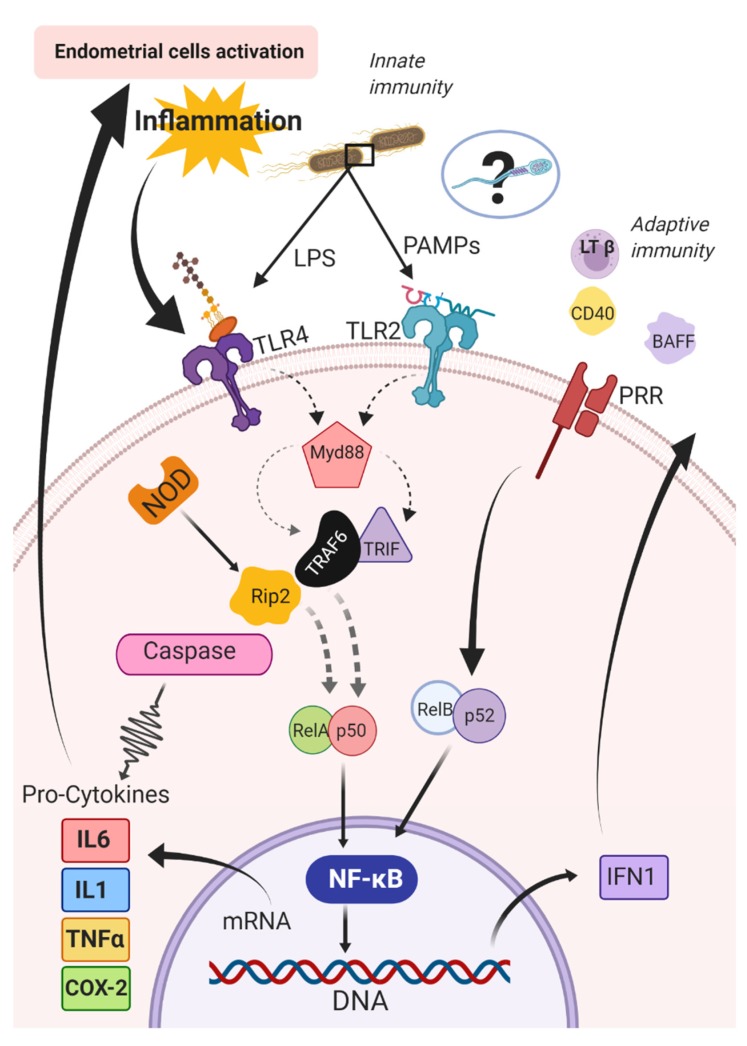
Canonic and alternative pathways for activation of the nuclear factor kappa beta (NF-kB) in the endometrium of mares. BAFF: B cell activating Factor; CD40: Cluster of differentiation 40; COX-2: cyclooxygenase-2; IFN1: type 1 interferons; IL1: interleukin 1; IL6: interleukin 6; LPS: lipopolysaccharides; LTβ: lymphotoxin β; MyD88: Myeloid differentiation primary response 88; NF-κB: nuclear factor kappa beta; NOD: nucleotide-binding and oligomerization domain; PAMPs: pathogen-associated molecular patterns; PRR: pattern recognition receptors; RelA/p50 and RelB/p52: subunits of the NF-κB complex; Rip2: receptor-interacting protein 2; TLR2: Toll-like receptors type 2; TLR4: Toll-like receptors type 4; TNFα: tumor necrosis factor-alpha; TRIF: TIR-domain-containing adapter-inducing interferon-β; TRAF6: receptor-associated factor 6.

Pattern recognition receptors can respond to a diverse array of antigens and then activate pro-inflammatory cytokines [157,158,159] to regulate the immune response [160,161]. Activation of TLRs is a key event in the initiation of the inflammatory cascade [148], which stimulates nuclear factor kappa beta (NF-κB). The nuclear NF-κB is composed of five subunits, (RelA {p65}, RelB, ReL, p50, p52) and can be activated by innate (i.e., canonic) or adaptive (i.e., alternative) immune pathways [162]. The canonic pathway is triggered by microorganisms and pro-inflammatory cytokines (e.g., IL1 and TNFα) [162]. The triggering of this pathway entails the activation of RelA- or cRel- [163], which are regulated by IkB kinase β (IKKβ) through phosphorylation of inhibitors of kB (IkB) [164]. The IkB kinases are the key regulators of the NF-κB pathway [165]; conversely, in the alternative pathway, NF-κB is activated by other bioproducts such as lymphotoxin β, CD40 ligand, B cell-activating factor, and receptor activator of NF-κB [166,167,168,169,170,171]. The activation of RelB/p52 complex and IkB kinase α is required in this pathway for phosphorylation and processing of the precursors p52 p100 [166] (Figure 2).

The NF-κB pathway then activates genes coding for pro-inflammatory cytokines, including chemokines and cyclooxygenase-2 (COX-2) [148,172]. Cytokines and COX-2 signal the immune cells modulating the acute inflammatory response [173]. Gene expression of members of both NLR and TLR families are upregulated after intrauterine inoculation with *Escherichia coli* in mares. In addition, several immune-related signal transduction pathways, including the mitogen-activated protein kinase, the NF-κB and TNFα signaling pathways, and the pathway for cytokine–cytokine receptor interaction, were also enriched after inoculation with *Escherichia coli* [29]. It should be noted that it remains to be determined if sperm induces a similar immune response to microorganisms.

A subset of these PRRs requires simultaneous binding to other endogenous cell surface receptors to be activated. As an example, when TLR4 binds to CD14, the subsequent detection of LPS alters downstream inflammatory responses [138,172]. After antigen recognition, the recruitment of adaptor proteins together with the MyD88-dependent cascade leads to the secretion of pro-inflammatory cytokines or the TIR-domain-containing adapter-inducing interferon-β (TRIF)-dependent cascade, which results in the production of type 1 interferons (IFN) in addition to inflammatory cytokines including chemokines [138,172]. The MyD88-dependent cascade induces the IL-1R-associated kinase family of kinases and consequently, activation of the ubiquitin-dependent kinase by TNFR-associated factor 6 [174,175]. The ubiquitin-dependent kinase induces NF-κB activation and the induction of the non-specific immune response by NF-κB transcription [176].

Initially, cytokines are synthesized as pro-molecules needing to be activated. Many types of molecules can activate cytokines (e.g., elastase, cathepsins, metalloproteinases, and trypsin). However, caspases, a large family of evolutionarily conserved proteases, play this role more broadly than other molecules [177]. For instance, the caspase-1 family (i.e., caspase-1, -4, -5, -11, -12, and -14) is primarily involved in the regulation of cytokines activation [177], and the caspase-3 family (e.g., caspases -3 and -7) can activate pro-inflammatory cytokines [178,179,180]. Specifically, caspase-1 activates IL1β, which is constitutively expressed and up-regulated in the endometrium of mares after experimental bacterial inoculation [29] and is also synthesized by NF-κB stimulation [173]. Under the action of prostaglandin-endoperoxide synthase, especially COX-2 during inflammation, prostaglandin synthesis occurs [181]. In the horse, an increase in COX-2 expression has been noted in the endometrium after exposure to seminal plasma or extender [182], as well as a local increase in prostaglandin F2 alpha (PGF2α) concentration in the uterus of normal mares 16 h after breeding [30].

From the synthesis of prostaglandins and pro-inflammatory cytokines, mainly interleukin 1 (IL1), interleukin 6 (IL6), and tumor necrosis factor-alpha (TNFα), vascular endothelial cell activation occurs. This leads to a constriction of the arterioles and dilation of the venules at the affected site, thereby increasing vascular permeability and exudate leakage to the interstitium, causing local edema [183]. The endometrial expression of various pro-inflammatory interleukins, including interleukin 1β (IL1β), chemokine ligand 8 (CXCL8, formerly known as IL8), and TNFα, is higher in susceptible mares to PBIE than in resistant mares, even before exposure to an antigen. This increase in susceptible mares is also noted following challenges with pathogens or sperm [31,182,184].

**Figure 3:**
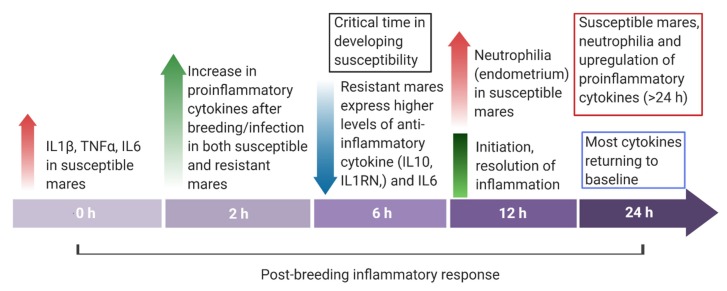
Overview of the endometrial cytokine dynamics in mares resistant and susceptible to endometritis from immediately before (0 h) to 24 h post-breeding.

With alterations in the permeability of the vascular endothelium, cellular responses begin. Vascular endothelial cells increase in the expression of P-selectin via the inflammatory stimulus, which binds to L-selectin on the surface of neutrophils, inducing chemotaxis [185]. Then, neutrophils produce integrins to bind to adhesion molecules on endothelial cells until complete arrest and adherence to the walls of blood vessels [186]. Following the detection of foreign material, neutrophils migrate from the endometrium to the uterine lumen within 30 minutes [11] and have a peak inflammatory response between 6 and 12 h later [2] (Figure 3). Mares susceptible to PBIE experience increased neutrophilia at 2 and 12 h after breeding in comparison with resistant mares [16]. In addition to phagocytosis, neutrophils also secrete additional cytokines and chemotactic mediators, further inducing inflammation [186]. Leukocytes then release prostaglandins, which promote myometrial contractility and assist with the physical clearance of the uterus in the healthy mare [187].

Neutrophils are the first immune cell line to respond after antigen recognition by innate immunity. In addition to phagocytosis and the release of lytic enzymes in response to antigens and pathogens, neutrophils form neutrophil extracellular traps (NETs). Neutrophil extracellular traps are DNA-associated molecules with antimicrobial and immunomodulating properties [188], which are induced by different inflammatory agents, such as reactive oxygen species [189], antibody-antigen complexes [190], CXCL8, lipopolysaccharide, and phorbol-myristate-acetate [191]. The antimicrobial activity of NETs, called NETosis, is due to neutrophils rupture and release of granules, allowing their chromatin to come in contact with antigens and other immune cells [192]. Several enzymes (e.g., elastase, protein 3, cathepsin G, and myeloperoxidase) and histones play a role in NETosis [193,194,195,196]. The formation of NETs is a complementary mechanism to eliminate bacteria, which may cause endometritis in mares [197].

When exacerbated, this increase in pro-inflammatory signals and the recruitment of immune cells can lead to tissue damage. Therefore, mechanisms are necessary to terminate the process for the resolution of the inflammatory process. An increase in anti-inflammatory or pleiotropic cytokines is noted as quickly as 2 to 6 h after breeding in the resistant mare [16] (Figure 3). They enact their properties by inhibiting the production of pro-inflammatory mediators, competing for pro-inflammatory receptors, or causing cell death [198]. Interleukin-10 (IL10), -1R antagonist (IL1RN), -4 (IL4), and -13 (IL13) are all considered anti-inflammatory and play an important role in the termination of this inflammatory response [14,199,200,201]. It has been well documented that IL1RN plays a role in balancing pro- and anti-inflammatory effects, because this cytokine competes with IL1 for binding to IL1 receptors, which prevents the binding of IL1α and IL1β [202]. Typically, IL10 is synthesized relatively late in the inflammatory response and acts as a generalized anti-inflammatory effector by reducing the transcription of pro-inflammatory cytokines by monocytes and macrophages [203,204].

Additionally, while IL6 is initially pro-inflammatory in response, its function is pleiotropic due to its ability to activate varying receptors and pathways to function as anti-inflammatories later in the inflammatory process. A study found the initiation of these anti-inflammatory cytokines to be defective in the susceptible mare [16]. At 6 h post-challenge with sperm, the endometrium of the resistant mare experienced an increase in IL10, IL1RN, and IL6 expression [16]. Susceptible mares had a significantly lower expression of these cytokines, indicating a failure to induce their response [184]. The authors hypothesized that this failure to mount an anti-inflammatory response leads to a prolonged endometrial inflammatory response in the mare susceptible to PBIE.

In addition to a defective anti-inflammatory cytokine production, susceptible mares have also been reported to have impaired myometrial contractility. A study found mares susceptible to PBIE to have a different myometrial response to the bacterial challenge when compared with the resistant mare, and this included the frequency, duration, and intensity of the contractions [205]. Interestingly, myometrial contractility is strongly connected to signaling within the immune system and particularly in the cytokine induction of nitric oxide (NO). Produced by inducible nitric oxide synthase, NO is a calcium-independent widespread signaling molecule that induces smooth muscle relaxation [206,207]. Interestingly, pro-inflammatory mediators, such as IL1 and IFNα, lead to increased transcription of this molecule [206]. This reduced smooth muscle activity is thought to interfere with the uterine clearance of mares susceptible to PBIE [10].

Numerous studies have investigated the role of NO in susceptibility to equine endometritis. Studies conducted with equine endometrial explants indicated a dose-dependent response to stimulation with NO, although a decreased response was noted in samples with lower endometrial quality [208]. In the susceptible mare, both NO expression and activity are upregulated [10,35,209]. The prolonged and undeterred increase in pro-inflammatory cytokines, such as IL1β, may be the cause of this increase in NO activity, leading to smooth muscle relaxation and decreased myometrial activity, all of which contribute to the pathophysiology of PBIE.

The stage of the estrous cycle (i.e., high vs. low progesterone concentrations) also plays a role in the uterine immune response in mares [210]. During progesterone dominance, the equine uterus is highly susceptible to infection, whereas, under estrogen dominance, the uterus is more capable of clearing infections [210]. For instance, expression of serum amyloid A (SAA) and the anti-inflammatory IL10 increased 3 h after bacterial inoculation in diestrus, but it did not in estrus [30,211].

Interestingly, matrix metalloproteinases (MMP) types 2 and 9 are significantly up-regulated 5 h after inoculation with *Streptococcus zooepidemicus* in both estrus and diestrus [212]. Matrix metalloproteinases are involved in extracellular matrix (ECM) remodeling [213] and modulated by tissue inhibitors of MMPs (TIMPs), which also have been identified in the endometrium of mares and women [212,214]. The activity of TIMP-2 decreases during induced endometritis in mares [212]. The fine balance between TIMPs and MMPs has been suggested to play a role in the development of endometrial fibrosis and degeneration in mares [212], but this is still controversial [215]. It is worth noting that mares susceptible to PBIE typically display endometrial fibrosis and other degenerative changes such as modifications in the type of collagen deposition [216].

The pathogenesis and etiology of endometrial fibrosis in mares remain poorly understood. It is known that MMPs and TIMPs regulate collagen deposition and other components of the ECM associated with fibrosis [217,218,219,220]. Also, NET components (e.g., myeloperoxidase, elastase, and cathepsin G) also up-regulate collagen types 1 and 3 and transforming growth factor-β1 [221,222,223]. In addition, endometrial expression of IL1β and IL6 is up-regulated during the progression of endometrial fibrosis in mares [224]. *In vitro* studies have shown the profibrotic effects of IL1β in the endometrium and other tissues [220,225,226]. A recent *in vitro* study suggested that IL1β and IL6 modulate ECM, MMP, and TIMP production in equine endometrium cells and might be important regulators in the pathogenesis of fibrosis [227]. Apparently, there might be an association between inflammation and development of endometrial fibrosis, in which IL1β and IL6 increase expression of ECM components, such as MMPs [227].

Additionally, endometrial epithelial cells produce antimicrobial peptides (e.g., defensins, elafin, cathelicidin, lactoferrin, and lysozyme), allowing for the nonspecific degradation of microbes and sperm alike [228]. In addition to their microbiocidal activity, these proteins affect cytokine induction, chemotaxis, and cell proliferation and modulate both innate and acquired immunity [211]. Antimicrobial peptides are modulated by the presence of bacteria, stage of the estrous cycle, and inflammation. These include factors that destabilize bacterial cell walls, such as defensins, lysozyme, and secreted phospholipase A2 [229,230] and those that inhibit bacterial enzymes, such as secretory leukoprotease inhibitor, which is also known as equine neutrophil antimicrobial peptide 2 [231,232,233]. Many of these antimicrobial proteins are increased in the endometrium of the susceptible mare population, including secretory leukoprotease inhibitor, equine β-defensin, lactoferrin, and lysozyme [28,234]. As many of these proteins are also produced within neutrophil granules, it is unknown if this increase is due to a defense mechanism within the endometrial glands of the susceptible mare or the increased neutrophilia caused by prolonged inflammatory processes.

Acute-phase proteins have also been investigated for their involvement and diagnostic capabilities in endometritis, as they are systemic markers of inflammation in the horse [235,236,237]. Serum amyloid A, haptoglobin, and fibrinogen have all been investigated [238]. The histologically normal endometrium of the mare has been shown to constitutively express mRNA for SAA at moderate levels [239]. Conflicting information exists regarding a detectable systemic SAA response to endometritis. One study found circulating SAA and fibrinogen to increase at 3- and 12-h post-inoculation with *Escherichia coli*, and this increase correlated with the endometrial expression of SAA [240]. In contrast, another study found no change in SAA concentrations following breeding with frozen/thawed semen [30]. Whether this difference was caused by the type of challenge used or due to the limited sample sizes remains to be determined. It should be noted that no difference in circulating SAA concentration or expression has been noted when comparing the susceptible to the resistant mare [199].

## 3. Diagnosis

**Figure 4:**
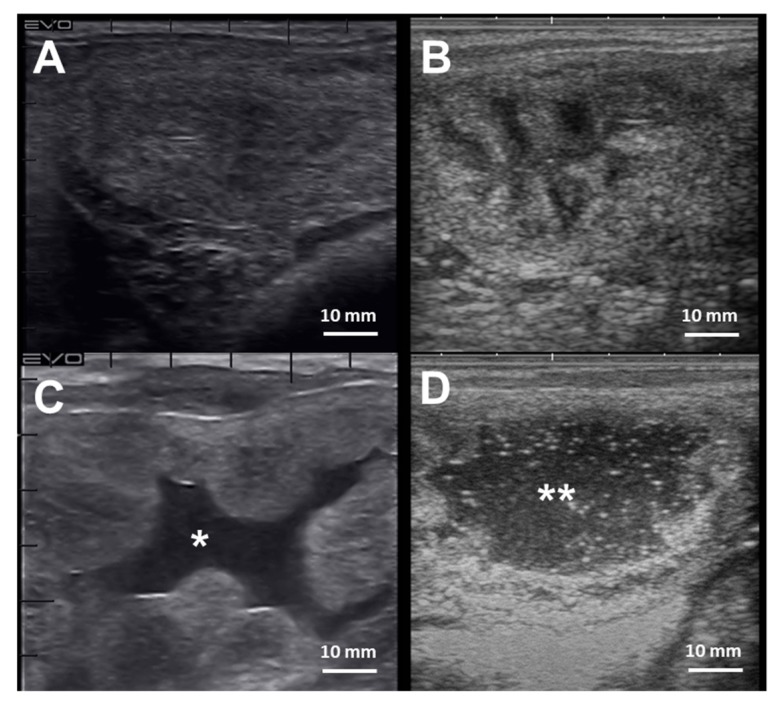
Cross-section ultrasonographic images of the uterine horns in mares: (**A**) An image from the equine uterus with no endometrial edema, or intraluminal fluid accumulation, typically seen in mares during diestrus; (**B**) the uterine horn of a mare in estrus, characterized by the presence of edema in the lymphatic vessels surrounding the endometrium submucosa giving the “orange-slice” aspect, (**C**) Exacerbated endometrium edema with extravasation and intraluminal fluid accumulation (*) in a mare with endometritis, and (**D**) extensive hyperechogenic intraluminal fluid accumulation (**) in a mare with endometritis. Scale bars 10 mm (**A–D**)

The diagnosis of endometritis entails a multi-modal approach coupled with a detailed clinical history. Endometrial culture, cytology, and biopsy are the most common tools employed to diagnose endometritis in mares [241] (Table 2). Mares susceptible to PBIE may have a history of accumulating intrauterine fluid before and after breeding (Figure 4), recurrent embryonic loss, early return to estrus, failure to become pregnant despite good breeding management, and presenting vulvar discharge.

**Figure 5:**
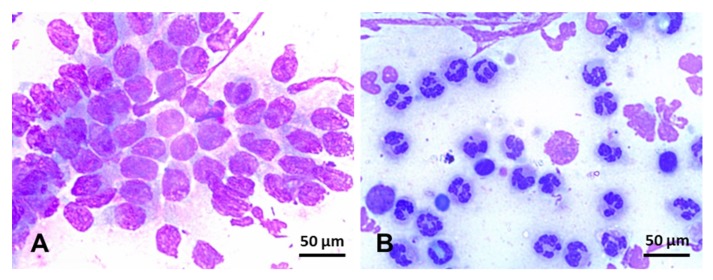
Endometrial cytology in mares stained with Romanowsky-type stain (×100). (**A**) Negative endometrial cytology; in this slide, endometrium epithelium cells, can be seen in the absence of inflammatory cells. (**B**) Positive endometrial cytology; few endometrium epithelium cells can be seen in this slide, but, conversely, hypersegmented neutrophils are overly represented in this smear. Scale bars 50 µm (**A** and **B**).

Endometrial cytology can be used to assess the type and proportion of inflammatory cells regarding endometrial epithelial cells present in the uterine lumen (Figure 5). In addition, cytology can occasionally detect the presence of bacterial colonies, hyphae, yeast, and urine crystals [242]. Specimens for endometrial cytology can be obtained with a simple or double-guarded cotton-tip swab, cytobrush, or low-volume uterine lavage [242,243,244]. Cytobrush and low-volume uterine lavages yield superior diagnostic samples than cotton-tip swabs [47,244] (Table 2). In addition, smears obtained with a cytobrush yield more cells (endometrial and PMNs) than cotton-tip swabs [47]. In addition, cytobrush smears had a positive association between the number of PMNs and the number of colonies of *β-hemolytic streptococci* [244] but not when *Escherichia coli* was the primary bacterial isolate [245]. After collection, endometrial smears are fixed and stained with Romanovsky-type stains for evaluation. Samples can be evaluated at 400x or 1000x magnification and quantified as the number of neutrophils for every 100 epithelial cells (EC) (Figure 5). The following categories may be used to define the endometrial inflammation: normal (no white blood cells (WBC) to rare WBC/100 EC), mild inflammation (1–2 WBC/EC), moderate inflammation (3–5 WBC/EC), and severe inflammation (>5 WBC/EC) [242]. As noninfectious endometritis will still lead to chemotaxis of neutrophils into the uterine lumen, cytologic examinations cannot be used singularly to rule out infectious endometritis. Therefore, samples for endometrial culture should always be harvested and interpreted in conjunction with cytology results.

Endometrial culture should always be collected before any uterine or vaginal procedure to avoid potential contamination. Endometrial culture can be done via double-guarded cotton-tip swab, low-volume uterine lavage, or biopsy [46,241,246,247,248]. Endometrial culture with double-guarded cotton-tip swab has lower sensitivity (0.34) in comparison with endometrial culture off a biopsy (0.82) [241] or low-volume uterine lavage (0.75) [245]. However, the specificity of low-volume uterine lavage (0.72) is reported to be lower than the other two techniques (swab—0.90; biopsy—0.90) [245]. After collection, the sample should be processed or placed in transport media (e.g., Ames or Steward) and kept refrigerated until arrival at the laboratory or streaked over culture media right way. In addition, to have microorganisms identified with biochemical tests or microbial proteins or genes, the growth can be grossly estimated by the number of colonies per plate as no growth (no colonies), very light (≤ 2 primary streak), light (3–5 primary streak), moderate (>5 into secondary streak), and heavy (>5 into tertiary streak) [33,249]. After bacterial or fungal isolation, an antimicrobial sensitivity test should be performed to determine the most suitable antimicrobial to treat the infection. In addition, PCR has been gaining popularity in clinical practice to identify bacteria and fungi in endometrium samples. Results can be available in 6 h, while the final culture and sensitivity results are pending [250].

**Figure 6:**
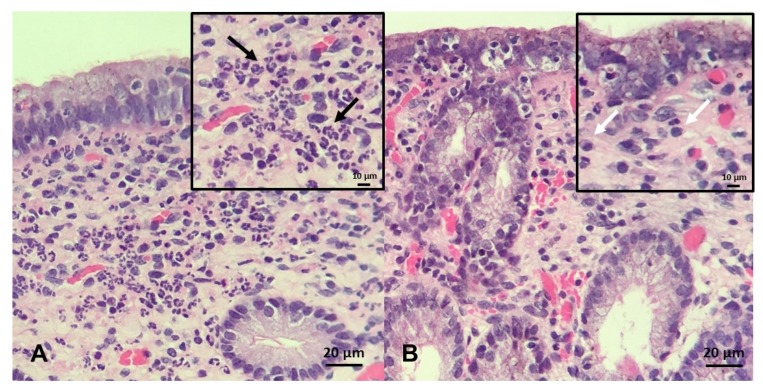
Endometrial biopsy specimens stained with H&E (×20 magnification, insert ×40): (**A**) A mare with acute, persistent breeding-induced endometritis characterized by a massive neutrophilic infiltrate in the stratum compactum and spongiosum; black arrows indicate neutrophils (**B**) a mare with chronic persistent breeding-induced endometritis characterized by heavy lymphocytic infiltrates present in the stratum compactum of the endometrium; white arrows indicate lymphocytes. Scale bars 10 µm inserts (**A** and **B**); and 20 µm (**A** and **B**).

The endometrial biopsy also can be used as a diagnostic tool for endometritis, as well as for a proxy for putative ability of mares carrying a foal to term [246,251,252,253]. A grading system was developed by Kenney and Doig, where the endometrium is assessed for glandular distribution, inflammatory cells, lymphatic lacunae, and fibrosis and then graded on a scale of I–III [254] (Figure 6). As these aspects of endometrial degeneration are associated with a predisposition to endometritis, a biopsy score combined with the mare’s clinical history can be used to predict fertility [251,253]. In addition, as mentioned above, the culture of biopsy samples improves sensitivity (0.82) when compared with double-guarded cotton-tip swabs (0.34) [246]. Recently, it has been proposed that gene expression in endometrial biopsies, such as equine β-defensin 1, lysozyme, and secretory leukoprotease inhibitor, can be used as a diagnostic test to identify mares susceptible to PBIE with overall sensitivities of 78-94% [28]; however, to date, these genes have yet to be tested in clinical practice.

## 4. Treatment

Traditionally, endometritis has been treated with uterine lavage, ecbolics, anti-inflammatories, and antimicrobials [20]. In recent years, the lack of response to traditional therapy and the increasing prevalence of antimicrobial-resistant pathogens has led to the development of alternative therapies to treat mares suffering from chronic endometritis [21]. Traditional and alternative therapeutic modalities are discussed herein.

### 4.1. Ecbolics

Ecbolics, are a pharmacological class of drugs used to stimulate uterine contractions to eliminate intrauterine fluid accumulation via the cervix and lymphatic drainage [255,256,257]. Oxytocin, the most commonly used ecbolic, is typically administered any time before breeding (except, up to 1 hour before breeding) and then between 4 h post-breeding and 72 h after ovulation. It is believed that sperm transportation to the uterine tubes is complete by 4 h post-breeding; thus, this is the shortest amount of time in which mares can receive an ecbolic or uterine lavage without compromising pregnancy rates [258].

After ovulation, cervical closure occurs, preventing the elimination of uterine fluid through the cervix [12]; however, minimal amounts of fluid can still be eliminated via lymphatic vessels present in the uterus. Smaller doses of oxytocin (10-20 units) induce rapid uterine contractions of short duration, whereas higher doses (e.g., 40 units) result in tetanic, less effective contractions [256,259,260,261]. The effects of exogenous oxytocin are expected to last ~45 minutes and therefore, requiring repeated doses [187].

Cloprostenol, a PGF2α analog, is another ecbolic used in clinical practice to treat intrauterine fluid accumulation in mares [21]. This drug induces longer myometrial activity (5 h) when compared to oxytocin [262,263], benefiting uterine cleansing and lymphatic drainage in mares with a pendulous uterus [263]. However, colic-like signs (e.g., pawing, abdominal contractions, sweating, tachypnea) following PGF2α are undesirable features [264]. In addition, administration of PGF2α starting 18–24 h post-ovulation can compromise corpus luteum formation and function, thus resulting in suboptimal systemic progesterone concentrations and decreased pregnancy rates function [262,265].

Carbetocin, an oxytocin analog, is another ecbolic drug used to promote milder and longer uterine contractions when compared with oxytocin [266,267]. Carbetocin is a cyclic octapeptide with a substitution in the amino group by a hydrogen atom with a modification of the disulfide bond by a thioether bond and substitution of the hydroxyl group of tyrosine by a methoxyl group, which helps to avoid early metabolization following administration [266]. Carbetocin has a 2.5-fold longer half-life (17 minutes) than oxytocin (6 minutes) [266,267]. However, to date, there is no study comparing the ability of oxytocin and carbetocin to promote uterine clearance in mares.

### 4.2. Antibiotics

While antimicrobials are necessary to treat infectious endometritis in mares, these drugs are also often used with no clear medical indication, an example of which is the common single antibiotic dose intrauterine infusion following breeding [8]. The irrational use of antimicrobials has led to the rapid development of antimicrobial resistance. Therefore, the proper identification of microorganism(s), in addition to sensitivity to antimicrobials, is paramount to treat endometritis and prevent the development of antimicrobial resistance successfully.

Because the most common bacterial isolates in the mare’s reproductive tract are *Streptococcus spp, Escherichia coli, Klebsiella sp, Pseudomonas sp,* and *Staphylococcus sp* [20,42,47,48,49], the most common antimicrobials used to treat endometritis include β-lactam (e.g., ceftiofur, ampicillin, penicillin) and aminoglycosides (i.e., gentamicin and amikacin) [268] (Table 3). It is interesting that *Streptococcus zooepidemicus* and *Escherichia coli*, the two most common isolates from mares with endometritis, were reported to be highly resistant to common antimicrobials [269].

The mechanisms through which bacteria become resistant to antibiotics have been characterized as modifications in the cell target that prevent antibiotic binding, synthesis of enzymes that digest the antimicrobial agent, activation of an alternate pathway that bypasses the mechanism of action of the antimicrobial drug, down-regulation or elimination of transmembrane porins from where drugs can access the cell, and elimination of drugs by efflux pumps [271,272]. Additionally, *Streptococcus zooepidemicus* can cause deep-seeded dormant infections (i.e., vegetative stage) in the uteruses of mares, making the bacteria resistant to conventional therapy [51]. One commercial product called b-Activate (Bojesen and Petersen Biotech, Copenhagen, Denmark) has been developed to stimulate dormant *Streptococcus* into an active proliferative stage, thus making the infection treatable with antimicrobials [52]. The cost of the product has limited its widespread use in clinical practice.

Antimicrobial usage for the treatment of endometritis can be administered either via intrauterine infusion or systemically. It should be noted that not all systemic antimicrobials can be administered in the uterus without some adjustment. An example of this is in fluoroquinolones such as enrofloxacin. The systemic formulation of enrofloxacin cannot be used in the uterus, as the vehicle causes endometrium necrosis [273]. However, if prepared with a different vehicle, enrofloxacin can be safely administered within the uterus [274]. In addition, aminoglycosides (e.g., gentamicin and amikacin) need to be buffered with sodium bicarbonate to counterbalance their acidic pH of 4 [20,275]. Infusion of incompatible antimicrobials into the uterus may precipitate and leave residues in the uterus, which could disturb embryo–endometrium interactions.

There is conflicting evidence regarding the most suitable route for antimicrobial administration to treat endometritis in mares. Intrauterine antimicrobial administration involves minimal systemic disturbances of the microbiome in other body systems, higher local endometrium concentrations, and a lower total amount of antimicrobial needed when compared with systemic administration [268,275]. It should be noted that systemic administration of antimicrobials, particularly for a prolonged period and when drugs need to be changed during treatment, can result in highly undesirable complications such as diarrhea, colitis, and systemic anaphylactic reactions [268]. In contrast, intrauterine administration may also irritate the endometrium and create noninfectious endometritis itself. Additionally, intrauterine antibiotics are presumed to lead to inconsistent tissue penetration, which favors the development of antimicrobial resistance. Presumably, deep-seeded and dormant endometrial infections, such as those caused by *Streptococcus*, may benefit from systemic administration, as intrauterine infusions may not result in deep penetration of antimicrobials.

Treatments for fungal endometritis are not well elucidated, as there is a lack of controlled studies on fungal endometritis in mares and the pharmacokinetic and pharmacodynamics of these drugs in the reproductive tract. The initial treatment should address the predisposing factors (e.g., poor perineal conformation, immunosuppression, discontinue intrauterine infusions with antibiotics) in combination with uterine lavages and ecbolic drugs. Elimination of predisposing factors for endometritis can sometimes restore the fertility of these mares [54,56]. However, when needed, three types of antifungal agents (polyenes, imidazoles, and triazoles) can be used to treat mares with fungal uterine infections [49,56]. The polyenes are considered fungicidal drugs, and the azoles (e.g., imidazoles and triazoles) are fungistatic [270]. Interestingly, polyenes and azoles have similar mechanisms of action, as they act in the fungal membrane by binding the ergosterol or inhibiting ergosterol biosynthesis [276], respectively (Table 4). *In vitro* results indicate that polyenes (e.g., amphotericin B, natamycin, and nystatin) are more effective in treating fungal endometritis than azoles (e.g., ketoconazole, fluconazole, and miconazole), mainly for molds with septated hyphae [49].

### 4.3. Uterine Lavage and Treatment for Biofilm

Uterine lavage is recommended in mares with excessive intrauterine fluid accumulation (e.g., >2 cm depth) and high ultrasonographic echogenicity [277]. Crystalloid solutions such as lactated Ringer’s solution (LRS) and 0.9% saline are most commonly used to lavage mares’ uteruses [278]. As studies have demonstrated that some bacteria, such as *Escherichia coli*, can utilize lactate (present in LRS) [279] and gluconate (present in Plasmalyte, a crystalloid solution less commonly used for uterine lavages) as substrates for growth [280], uterine lavage with crystalloids cannot be used alone to treat infectious endometritis. These solutions can be enriched with anti-septics (e.g., povidone-iodine and hydrogen peroxide), vinegar to change the uterine microbiome in cases of fungal endometritis, and additives to break biofilm such as mucolytics (e.g., N-acetylcysteine, dimethyl sulfoxide, ethylenediaminetetraacetic acid-2-amino-2-hydroxymethyl-propane-1,3-diol alone or in combination with Tris; disodium ethylenediaminetetraacetate dehydrate-2-amino-2-hydroxymethyl-1,3-propanediol). Despite the wide use of these products in the treatment of endometritis, it is unknown how they affect the resident uterine microbiome and how these agents can be used to restore the balance of microorganisms *in utero*.

Uterine lavage helps by physically removing microorganisms, debris, inflammatory cells and mediators, and dead sperm from the lumen, which can be detrimental for the sperm before breeding or the embryo after breeding [277,278,281]. It can be performed at any time before or starting four hours after breeding. Four hours after breeding is recognized as the minimal time necessary for sperm to reach the uterine tubes, not interfering with fertility [258,282,283]. Additionally, in a stagnant inflammatory state, the use of uterine lavage can reintroduce viable neutrophils to reinstate an active degradation of microbes.

### 4.4. Immunomodulatory Agents

The treatment of PBIE with non-steroidal anti-inflammatory drugs (NSAIDs) is still controversial, as inhibition of prostaglandin-endoperoxide synthase (types 1 and 2) and therefore the arachidonic acid cascade is a primary effect. This would decrease PGF2α production, potentially diminishing myometrial activity and leading to delayed uterine clearance. Two studies on phenylbutazone and flunixin meglumine (non-selective COX-2 NSAIDs) observed delayed uterine clearance and an increased inflammatory reaction in mares receiving treatment [284,285]. Additionally, the administration of high (>2X labeled dose) and sustained doses of NSAIDs in pre-ovulatory mares has been shown to increase the rate of anovulatory hemorrhagic follicles [286,287]. However, the labeled dose of NSAID did not interfere with ovulation [288]. In addition, the combination of NSAID with oxytocin has been shown to improve uterine clearance [255], reduce PMNs infiltration, and decrease endometrial expression of COX-2 [289] in susceptible mares.

Another alternative is the selective COX-2 NSAIDs, which do not act on the cyclooxygenase-1 (COX-1) pathway [290]. Firocoxib, a COX-2 selective NSAID, has been described to mitigate post-breeding inflammatory response in mares with a reduction in COX-2 in the endometrium of mares treated during the periovulatory period, while not affecting ovulation rates [291]. Vedaprofen, another selective COX-2 inhibitor, has been reported to affect fertility in mares with PBIE positively but without interfering with intrauterine fluid accumulation and the inflammatory score of the endometrium [292]. Based on these results, selective COX-2 NSAIDs may be an alternative treatment to mares suffering from PBIE.

Glucocorticoids are routinely used to modulate the post-breeding inflammatory response in mares. The seminal study used multiple prednisolone doses before ovulation for mares being bred with frozen semen [136,293]. A posterior study demonstrated that administration of dexamethasone before breeding reduced endometrial inflammation and improved pregnancy rates in barren mares with a history of or presenting predisposing signs for PBIE [15].

On a molecular level, dexamethasone affects the endometrial expression of cytokines and SAA in susceptible mares after intrauterine inoculation with *Escherichia coli* [33] or sperm [34,294]. A reduction of pro-inflammatory IL1β, CXCL8, and SAA and suppression of inflammatory mediators, such as COX-2, lipo-oxygenase 5, and NO, were reported after dexamethasone therapy, while an increase in the inflammatory modulating and anti-inflammatory IL6, IL10, and IL1RN was also observed after treatment [33]. Additionally, the administration of dexamethasone altered the production of acute-phase proteins following bacterial challenge [295] and did not alter the phagocytic function of blood-derived PMNs [296], which could predispose the mare to a secondary infection. This alteration in the acute phase protein profile has also been noted following treatment with prednisolone [297]. A study found that the administration of prednisolone following inoculation with bacteria to increase antitrypsin and transthyretin while reducing IgG [297]. It should be noted that while glucocorticoid therapy is associated with reduced endometrial edema following breeding, repeated administration of either dexamethasone and prednisolone may suppress the luteinizing hormone (LH) surge and increase the rate of ovulation failure [298]. In one study, prolonged administration (5 days) of dexamethasone tended to reduce (40%) ovulation rates in mares when compared with placebo (100%), while the prolonged administration of prednisolone did not alter these results (83%) [298]. In horses, dexamethasone may affect hypothalamic GnRH and pituitary LH secretion [299,300], and in other species, glucocorticoids may alter hypothalamic [301], anterior pituitary [302], and ovary [302,303] function. Therefore, a single and low-dose injection is recommended for the treatment of post-breeding endometritis in mares rather than repeated doses.

The use of bacterial extracts has also been found to alter the immune response to endometritis [31,32,33,184,304,305]. Mycobacterium phlei cell wall extract (MCWE) is a commercial immunomodulator (Settle, Bioniche Animal Health, Athens GA, USA) that is used for the treatment of equine endometritis caused by *Streptococcus zooepidemicus*. This immunomodulator acts by enhancing the innate humoral immune response. It has been shown to decrease the endometrial expression of pro-inflammatory cytokines IL1β, IL6, and TNFα in susceptible mares following both breeding and challenge with Gram-positive bacteria, while also increasing the expression of the anti-inflammatory IL10 [32]. In addition, mares treated with MCWE also show a decrease in NO [36]. Although MCWE did not affect endometrial cytokine expression following challenge with *Escherichia coli*, a pronounced reduction in bacterial growth was noted following treatment, in addition to a reduction in intrauterine fluid accumulation [33]. This was confirmed in the following study, and MCWE was found to be bactericidal regardless of being administered intravenously or in the uterus [304].

Another immunostimulant that has been described to be able to improve pregnancy rates in mares is the *Propionibacterium acnes* (EQStim, Neogen Corp, Lexington KY, USA) [305]. This therapy induces a non-specific cell-mediated response, predominantly by macrophage activation and cytokine release. In the single study investigating this immunostimulant, mares with clinical endometritis were treated with intravenous *Propionibacterium acnes* [305]. Repeated administration of this treatment as an adjunct to conventional therapies has improved pregnancy rates and increased live foaling rates in mares with a cytologic diagnosis of endometritis in comparison with a placebo. Unfortunately, the molecular mechanisms of using this therapy have yet to be investigated.

### 4.5. Lactoferrin

Lactoferrin is an 80kDa protein found promiscuously throughout the body, including in both the reproductive and immune systems [306]. It is believed to be bactericidal due to its ability to chelate free iron [307]. The endometrial expression of lactoferrin varies depending on the stage of the estrous cycle and is increased during estrus, indicating its endocrine dependency [234]. The administration of recombinant lactoferrin at the time of breeding in the normal mare has shown varying results, with one study finding no change in cytokine expression [308], while another found a significant decrease in endometrial IL6, alongside a trend towards a decrease in CXCL8, IL1β, and TNFα [309], indicating its anti-inflammatory properties.

Interestingly, this decrease in TNFα was also noted in mares susceptible to PBIE when lactoferrin was administered at the time of breeding [310]. When administered 6 h post-breeding in susceptible mares, lactoferrin decreased the expression of pro-inflammatory interferon gamma while increasing anti-inflammatory IL1RN [311]. This study also found varying concentrations of lactoferrin (50–500 µg) to decrease PMN infiltration in the susceptible mare, although it did not affect uterine fluid retention. The authors suggested that the recommended dose for human recombinant lactoferrin intrauterine infusion should be 1mL (50 μg/mL) diluted in 10 mL of LRS, which is comparable to the average concentration noted in the equine ejaculate. Interestingly, in other species, lactoferrin is administered for its bactericidal and anti-biofilm properties [312,313], but these findings have not been investigated in the horse.

### 4.6. Platelet-Rich Plasma

Platelet-rich plasma (PRP) is whole blood plasma with concentrated platelets (3-5-fold). It has been commonly used in equine clinical practice to treat joints, bursae, and soft tissue injuries (e.g., tendonitis, tenosynovitis, and skin wounds) [314,315,316,317]. Additionally, the combination of autologous plasma to antibiotic therapy has been reported to improve pregnancy rates for lactating and barren mares [318].

The biological mechanisms of PRP on the inflammatory response are not yet well elucidated. However, some studies [319,320,321,322] have demonstrated an anti-inflammatory action of PRP due to its ability to suppress the expression of COX-2, metalloproteinase-3 (MMP-3), TNFα, IL1, and vascular adhesion molecules [323]. Additionally, platelet granules contain antimicrobial peptides (RANTES, platelet factor 4, and thymosin beta-4), and these peptides may contribute to PRP’s known bactericidal activity against *Staphylococcus aureus*, *Escherichia coli*, and *Klebsiella pneumoniae* in the human [324,325,326,327,328,329,330,331]. All these bacteria are known causes of endometritis in mares [20].

For the treatment of endometritis in the horse, one study found that PRP administration at the time of breeding decreased the intrauterine inflammatory response in mares suffering from chronic endometritis, although it did not affect NO production [332]. This concurs with another study reporting that PRP decreased endometrial expression of COX-2, decreased PMN numbers in the uterine lumen, and increased pregnancy rates [333]. It has also been shown to act as an anti-inflammatory, and treatment of susceptible mares led to a down-regulation of endometrial IL1β, IL6, and CXCL8 expression [332,333,334,335].

### 4.7. Stem Cells

The use of mesenchymal stem cells (MSCs) has rapidly gained interest in human and veterinary medicine to modulate the inflammatory processes [336,337]. These cells can differentiate into skeletal myoblasts, renal parenchyma, hepatic epithelium, gut and skin epithelia, neuroectodermal cells [338], and even endometrial cells [339]. They can also signal residual cells through anti-apoptotic, chemotactic, and immune-modulatory properties and can be harvested from variable tissues with differential potency.

Injection [340] or infusion [341] of MSCs has been described as a possible alternative for the treatment of endometrial fibrosis. Therapy with MSCs can induce an early (7 days) and also a prolonged (60 days) remodeling of the endometrium of mares suffering from chronic-degenerative endometriosis by modulating the expression patterns (e.g., cytokeratin, vimentin, α-smooth muscle actin, and laminin) associated with the development of pathological fibrosis in the horse endometrium, as well as promoting glandular epithelial cells proliferation [341]. A report found that MSC administration decreases neutrophil numbers and increases anti-inflammatory ILRN expression in the endometrium of the normal mare [342]. Interestingly, while one study found that MSCs also attenuate markers of uterine inflammation following treatment, cells were unable to penetrate the endometrium and remained within the uterine lumen [343]. However, another study reported that intrauterine infusion of MSCs effectively engrafted MSCs in periglandular spaces [344]. In addition, it is known that MSCs can up-regulate anti-inflammatory cytokines (e.g., IL2, IL4, IL10 and basic fibroblast growth factor) and down-regulate pro-inflammatory cytokines (TNFα, IL1β and IL17) in animal disease models and rats within the endometrium [345,346,347]. Using rats as an experimental model, some authors indicated a regeneration of endometrial cells or a protective effect against cell damage in rat’s endometrium using an intrauterine MSCs therapy by the higher expression of cytokeratin and vimentin, a marker of endometrial cells [345]. In addition, in that study, intrauterine MSCs therapy was able to up-regulate markers for endometrial receptivity (integrin agb3 and leukemia inhibitory factor) [345], which are regulators of endometrial function and have an important role in embryo implantation [348]. To date, no studies have evaluated the efficacy of MSCs in mares susceptible to PBIE, so additional work is necessary. In addition, it is worth noting that primates and rodents undergo decidualization (i.e., morphological and functional changes of the endometrium in preparation for pregnancy), and horses do not; thus, findings obtained in these species cannot necessarily translate into similar findings in horses.

## 5. Conclusions

Endometritis is the most important cause of subfertility in mares. Mares susceptible to PBIE are frequently older, have an impaired uterine immune response, and deficient physical barriers and other mechanisms of defense against infection. An imbalance of pro- and anti-inflammatory mechanisms plays a pivotal role in the immunopathogenesis of PBIE in mares. Infections of the endometrium can be established if the natural mechanisms (physical and molecular) of defense are compromised. In addition, the pathobiology of microorganisms associated with endometritis in mares has been poorly understood, along with the role of the resident uterine microbiome in preventing infection and how diseases and treatment alter the composition of these microbe populations. Traditional and non-traditional therapies for endometritis are based on reestablishing the natural mechanisms of defense (e.g., fixing some reproductive seals of the reproductive tract or immunomodulate inflammation). The current trend of preventing indiscriminate use of antimicrobials in animals and humans will benefit from the continued attempts to develop alternative antibiotic-free therapies to treat endometritis.

## Figures and Tables

**Table 1:** Common bacteria and fungi isolated from the uteruses of mares suffering from endometritis. G+: Gram-positive; G-: Gram-negative.

	Microorganisms	Superfamily	Features
**Bacteria**	*Streptococcus zooepidemicus*	Lactobacillales	G+, opportunistic agent, potentially venereal.
	*Escherichia coli*	Enterobacterales	G-, opportunistic, facultative anaerobic
	*Pseudomonas aeruginosa*	Pseudomonadales	G +, potentially venereal, aerobic
	*Klebsiella pneumoniae*	Enterobacterales	G-, opportunistic agent, facultatively anaerobic, potentially venereal
	*Staphylococcus spp*	Bacillales	G+, opportunistic, facultative anaerobic
	*Taylorella equigenitalis*	Burkholderiales	G-, venereal, microaerophilic, cause severe purulent endometritis
	*Enterobacter cloacae*	Enterobacterales	G-, opportunistic, facultative anaerobic
	*Proteus spp*	Enterobacterales	G-, opportunistic, anaerobic
**Fungus**	*Candida spp.*	Saccharomycetales	Yeast, 58–69% of fungal endometritis
	*Aspergillus spp.*	Eurotiales	Mold with septate hyphae, 25–26% of fungal endometritis
	*Mucor spp.*	Mucorales	Mold with aseptate hyphae, 5–12% of fungal endometritis

Adapted from Canisso and Coutinho da Silva [20], Beltaire et al. [49], and Coutinho and Alvarenga [53].

**Table 2:** Summary of common tools used to diagnose endometritis in mares.

Technique	Approach and Applications	Limitations
Ultrasound	Used as a screening tool to detect the presence, amount, and appearance of IUF, which can be suggestive of endometritis.	Not all mares affected by endometritis, particularly chronic endometritis, accumulate IUF. The amount and echogenicity of fluid can be useful to direct the need for additional diagnostic techniques and therapeutic regimens.
Cotton-tip swab	Fast, user-friendly, and inexpensive approach to collect samples for culture and cytology. Results in combination with cytology can be used to dictate therapeutic approaches	Only a small segment of the uterus is sampled, and thus, focal infections not generating a diffuse endometrial response can be missed. In comparison with cytobrush, fewer cells are recovered, and cells are slightly compressed, making the evaluation more difficult
Cytobrush	Fast, user-friendly, and inexpensive approach to collect samples for culture and cytology, although it is more commonly used for cytology.	Only a small segment of the uterus is sampled, and thus, focal infections can be missed. Bacteria in biofilm may not be detected.
Low-volume uterine lavage	The whole surface of the uterus can be sampled for culture and cytology, and thus this technique is more utilized for the diagnosis of challenge and chronic endometritis. The recovered fluid can be centrifuged or allowed to decant before cytological evaluation.	There is a risk of contamination with commensal microorganisms of the caudal reproductive tract. It requires at least one well-trained clinician and an assistant. An excessive amount of fluid can overdilute the sample and cause a false-negative and may challenge the cytological evaluation. Mares with a pendulous uterus can have poor fluid recovery.
Endometrium biopsy	While this approach is primarily used for histological evaluation, endometrium biopsy is a sensitive and specific approach to diagnose endometritis in mares by histological evaluation and culture of the biopsy. Particularly useful for deep endometrium infection. Results may guide the treatment strategies employed.	It requires a biopsy, which is a minor procedure but still invasive. It also requires well-trained laboratory personnel capable of performing cultures and histological evaluations

Adapted from Riddle et al. [40] and Canisso et al. [20]. IUF: intrauterine fluid accumulation.

**Table 3:** Common antimicrobials used to treat mares suffering from bacterial endometritis.

Drug Class	Therapeutics	Mechanism of Action	AMR
Aminoglycosides (e.g., amikacin sulfate, gentamicin sulfate, and neomycin)	Concentration-dependent, bactericidal, broad-spectrum, G-	Irreversible inhibition of bacterial protein synthesis by binding to the 30S subunit of the bacterial ribosome	Low incidence, efflux pumps, mutation at the drug binding site
Cephalosporin (β-lactam, third generation) (e.g., ceftiofur sodium, and ceftiofur crystalline-free-acid)	Time-dependent, bactericidal, broad-spectrum, G- and G+	Inhibition of cell wall synthesis by disruption of the peptidoglycan layer	Growing resistance based on PBP mutation-reduced permeability and enzymatic inactivation by β -lactamase. Most G- rods can produce β-lactamase
Fluoroquinolones (e.g., enrofloxacin, and ciprofloxacin)	Concentration-dependent, bactericidal, broad-spectrum, G- and some G+	Inhibits DNA gyrase (topoisomerase II) and topoisomerase IV	Mediated by target mutations in DNA gyrase
Extended-spectrum penicillins (β-lactam) (e.g., Ampicillin, and Ticarcillin)	Time-dependent, bactericidal, broad-spectrum, G+ and some G-	Interference in bacterial cell membrane synthesis by inhibition of the transpeptidases and peptidoglycan enzymes	Acquired resistance in G- by plasmid- or integron-mediated
Penicillins (natural β-lactam) (e.g., K penicillin, Na penicillin, and G Procaine)	Time-dependent, bactericidal, broad-spectrum, G+	Lysis of cells weakened by the loss of the peptidoglycan layer in the membrane by binding the PBPs in the outside of the bacteria wall	Mutation of PBPs that reduces bacterial permeability, and production of β-lactamase
Polymyxins (e.g., polymyxin B)	Concentration-dependent, bactericidal, broad-spectrum, G- (e.g., *Pseudomonas* spp).	Disorganize the membrane by binding LPS, disrupting the cell wall membrane, and increasing cell permeability by detergent-like action	Rare; modification of the LPS in the bacterial membrane and development of an efflux pump/potassium system
Sulfonamides (e.g., sulfamethoxazole) associated with pyrimidine (trimethoprim)	Time-dependent, bacteriostatic, broad-spectrum, G- and G+ (*Streptococcus* spp)	Interference in the biosynthesis of folic acid by competition with PABA for dihydropteroate synthetase	Mediated via a chromosomal mutation causing the hyper-production of PABA or insensitive dihydropteroate synthase
Nitroimidazole (e.g., metronidazole)	Concentration-dependent, G+ anaerobes		

Adapted from Giguère [270]. AMR: antimicrobial resistance; PABA: para-aminobenzoic acid; PBPs: penicillin-binding proteins; LPS: lipopolysaccharides.

**Table 4:** Common anti-fungal drugs used to treat mares suffering from fungal endometritis.

Drug Class	Therapeutics	Mechanism of Action	AMR
Polyenes (e.g., amphotericin B, natamycin, and nystatin)	Fungicidal or fungistatic, broad-spectrum against *Candida spp, Aspergillus spp,* and *Mucor spp*	Binding to ergosterol in the membrane to disrupt the cell wall	Rare; the only mutant fungus enhances synthetic pathways for alternative sterols that replace ergosterol in the cell membrane
Imidazoles (e.g., clotrimazole, ketoconazole, miconazole)	Broad-spectrum activity against *Candida* spp	Inhibition of ergosterol synthesis in the fungal cell membrane by inhibiting the enzyme 14-α-demethylase, ultimately increasing cellular permeability and cell leakage	Resistance is found in filamentous fungi and after prolonged therapeutic regimens
Triazoles (e.g., fluconazole, itraconazole)	Potent anti-*Aspergillus* activity	Blockage of cytochrome P450–dependent enzyme C-14-α-demethylase (necessary for the conversion of lanosterol to ergosterol)	Resistance involves a single-point mutation in the cyp51A gene, which encodes for 14-α sterol demethylase

Adapted from Giguère [270] and Beltaire et al. [49].

## Abbreviations

ACP	Antigen-presenting cells
BAFF	B cell-activating factor
C1q	Complement component 1
C3a	Complement 3a
C3b	Complement 3b
C4a	Complement 4a
C4b	Complement 4b
C5a	Complement 5a
CD14 CD40	Cluster of differentiation 14 Cluster of differentiation 40
COX-1	Cyclooxygenase-1
COX-2	Cyclooxygenase-2
CXCL8	Chemokine ligand 8
EC	Epithelial cells
ECM	Extracellular matrix
EDTA	Ethylenediaminetetraacetic acid-2-amino-2-hydroxymethyl-propane-1,3-diol
FoxP3	Forkhead box protein P3
GnRH	Gonadotropin-releasing hormone
IFN	Interferon
IFNα	Interferon type I α
IgA	Immunoglobulin A
IgG	Immunoglobulin G
IgM	Immunoglobulin M
IL1	Interleukin1
IL1RN	Interleukin 1 receptor antagonist
IL10	Interleukin 10
IL13	Interleukin 13
IL17	Interleukin 17
IL1α	Interleukin 1alpha
IL1β	Interleukin 1Beta
IL4	Interleukin 4
IL6	Interleukin 6
LH	Luteinizing hormone
LPS LRS	Lipopolysaccharides Lactated Ringer’s Solution
MAC	Membrane attack complex
MCWE	Mycobacterium phlei cell wall extract
MMP-3	Metalloproteinase-3
MMPs	Matrix metalloproteinases
MSCs	Mesenchymal stem cells
MyD88	Myeloid differentiation primary response 88
NETs	Neutrophil extracellular traps
NF-κB	Nuclear factor kappa-B
NK	Natural killer cells
NLR	NOD-like receptors
NO	Nitric oxide
NOD	Nucleotide-binding and oligomerization domain
NSAIDs	Non-steroidal anti-inflammatory drugs
PABA PBIE	Para-aminobenzoic acid Persistent breeding-induced endometritis
PBPs PGF2α	Penicillin-binding proteins Prostaglandin 2α
PMNs	Polymorphonuclear neutrophils
PRP	Platelet-rich plasma
PRRs	Pattern recognition receptors
SAA	Serum amyloid A
TIMPs	Tissue inhibitors of MMPs
TLR2	Toll-like receptors type 2
TLR4	Toll-like receptors type 4
TLRs	Toll-like receptors
TNFα	Tumor necrosis factor-α
TRAF6	Receptor-associated factor 6
TRIF	TIR-domain-containing adapter-inducing interferon-β
WBC	White blood cells
